# Urinary excretion of amino acids and their advanced glycation end-products (AGEs) in adult kidney transplant recipients with emphasis on lysine: furosine excretion is associated with cardiovascular and all-cause mortality

**DOI:** 10.1007/s00726-021-03091-8

**Published:** 2021-10-24

**Authors:** Svetlana Baskal, Adrian Post, Daan Kremer, Alexander Bollenbach, Stephan J. L. Bakker, Dimitrios Tsikas

**Affiliations:** 1 Core Unit Proteomics, Institute of Toxicology, Hannover Medical School, Carl-Neuberg-Strasse 1, 30625 Hannover, Germany; 2 Division of Nephrology, Department of Internal Medicine, University Medical Center Groningen and University of Groningen, Groningen, The Netherlands

**Keywords:** AGEs, Cardiovascular risk, Glycation, Kidney, Mortality, Post-translational modification, Transplantation

## Abstract

Arginine (Arg) and lysine (Lys) moieties of proteins undergo various post-translational modifications (PTM) including enzymatic *N*^G^- and *N*^ε^-methylation and non-enzymatic *N*^G^- and *N*^ε^-glycation. In a large cohort of stable kidney transplant recipients (KTR, *n* = 686), high plasma and low urinary concentrations of asymmetric dimethylarginine (ADMA), an abundant PTM metabolite of Arg, were associated with cardiovascular and all-cause mortality. Thus, the prediction of the same biomarker regarding mortality may depend on the biological sample. In another large cohort of stable KTR (*n* = 555), higher plasma concentrations of *N*^ε^-carboxymethyl-lysine (CML) and *N*^ε^-carboxyethyl-lysine (CEL), two advanced glycation end-products (AGEs) of Lys, were associated with higher cardiovascular mortality. Yet, the associations of urinary AGEs with mortality are unknown. In the present study, we measured 24 h urinary excretion of Lys, CML, and furosine in 630 KTR and 41 healthy kidney donors before and after donation. Our result indicate that lower urinary CML and lower furosine excretion rates are associated with higher mortality in KTR, thus resembling the associations of ADMA. Lower furosine excretion rates were also associated with higher cardiovascular mortality. The 24 h urinary excretion rate of amino acids and their metabolites decreased post-donation (varying as little as − 24% for CEL, and as much as − 62% for ADMA). For most amino acids, the excretion rate was lower in KTR than in donors pre-donation [except for *S*-(1-carboxyethyl)-l-cysteine (CEC) and *N*^G^-carboxyethylarginine (CEA)]. Simultaneous GC–MS measurement of free amino acids, their PTM metabolites and AGEs in urine is a non-invasive approach in kidney transplantation.

**Supplementary Information:**

The online version contains supplementary material available at 10.1007/s00726-021-03091-8.

## Introduction

**Scheme 1:**
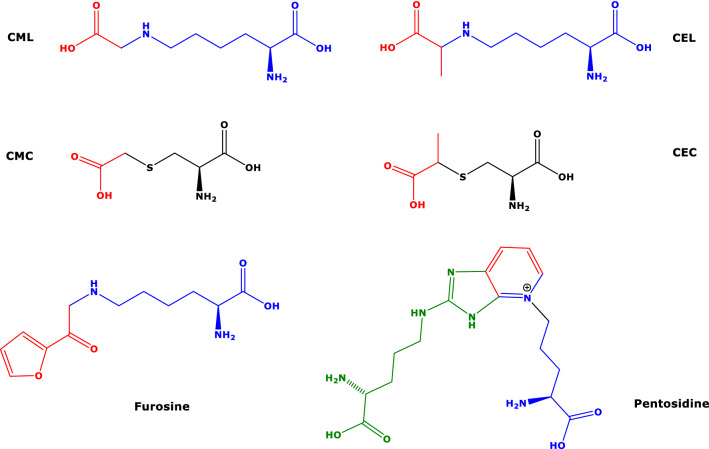
Chemical structures of selected advanced glycation end-products (AGEs) of l-lysine, l-cysteine and l-arginine. CML, *N*^6^-carboxymethyl-l-lysine; CEL, *N*^6^-carboxyethyl-l-lysine; CMC, *S*-carboxymethyl-l-cysteine; CEC, *S*-(1-carboxyethyl)-l-cysteine; furosine and pentosidine. The red-colored parts indicate the remaining of the glycation agent including glyoxal (in CML and CMC) and methylglyoxal (in CEL and CEC). Blue, black and green color indicates the residues l-lysine, l-cysteine and l-arginine, respectively

Amino acid residues in proteins undergo numerous enzymatic (e.g., citrullination and methylation) and non-enzymatic (e.g., glycation) post-translational modifications (PTMs). PTMs do not only alter the inherent biological activity of the native proteins, but may also be the origin of biologically active metabolites involved in renal and cardiovascular diseases (CVD). In proteins, the terminal guanidine (*N*^G^) group of l-arginine (Arg), the terminal amine (*N*^ε^ or *N*^6^) group of l-lysine (Lys), and the sulfhydryl (SH) group of l-cysteine (Cys) react with chemically reactive carbonyl groups such as those of reducing sugars (e.g., glucose, fructose, pentose) to form intermediate reaction products. The so-called early-stage products or Amadori products, such as hemoglobin A_1c_ (HbA_1c_), which is an established clinical marker for diabetes, can react further to finally form the so-called advanced glycation end-products (AGEs) (Nagai et al. 2014; see also Rabbani and Thornalley 2020; and Sibbersen and Johannsen 2020). AGEs are also formed in vitro during heating of carbohydrate-rich food. AGEs are often measured in blood and sporadically in urine, and in clinical trials they serve as markers for carbohydrate metabolism and protein denaturation (Nagai et al. 2014). The chemical structures of commonly measured AGEs are shown in Scheme 1. They include *N*^6^-carboxymethyl-l-lysine (CML), *N*^6^-carboxyethyl-l-lysine (CEL), *S*-carboxymethyl-l-cysteine (CMC), *S*-(1-carboxyethyl)-l-cysteine (CEC), furosine, i.e., the AGE of fructose and Lys, and pentosidine, i.e., the AGE of Lys and Arg.

Pentosidine in plasma and tissue has been early measured in kidney transplantation studies (Hricik et al. 1993, 1996) and has been associated with kidney function in transplant recipients (Slowik-Zylka et al. 2010). Several studies investigated the involvement of certain circulating AGEs and other biomarkers in chronic kidney disease (Busch et al. 2004, 2010; Stein et al. 2003), both in patients after kidney transplantation (Baumann et al. 2008; Crowley et al. 2013; Franke et al. 2003; Hartog et al. 2004, 2005, 2006) and in patients after heart transplantation (Heidland et al. 2004). As AGEs exert their biological activities via the soluble and membrane-bound receptor of AGEs, sRAGE and mRAGE, respectively, several groups investigated potential mechanisms of their action in renal transplant recipients (Gross et al. 2007; Kalousová et al. 2009; Liu et al. 2015). Life-factors such as smoking (Schiel et al. 2003) and effects of immunosuppressive therapy in kidney transplant recipients (Xu et al. 2017) have been also investigated in this context.

In a study of one of our groups involving 555 stable kidney transplant recipients (KTR), we found that plasma concentrations of CML and CEL were independently associated with long-term risk of cardiovascular mortality (Sotomayor et al. 2019). The plasma CML and CEL concentrations were determined by liquid chromatography–tandem mass spectrometry (LC–MS/MS) to be on average 1.8 µM and 1.0 µM, respectively, i.e., with a molar ratio of 1.8:1, indicating CML as the more abundant AGE in plasma. In another study, involving 681 stable KTR, we determined in plasma and urine samples the concentration of symmetric dimethylarginine (SDMA), asymmetric dimethylarginine (ADMA) and its major metabolite dimethylamine (DMA) as measures of symmetric and asymmetric methylation of Arg residues in proteins, respectively (Frenay et al. 2015a, b; Said et al. 2019a,b; Post et al. 2021). These studies indicated that Arg methylation, an abundant PTM in humans, is differently associated with cardiovascular mortality: higher plasma concentrations of ADMA were associated with a higher cardiovascular risk and mortality, while higher urinary ADMA excretion rates were associated with a lower cardiovascular risk and mortality. These observations indicate that the outcome of studies may depend upon the biological sample, i.e., plasma vs. urine. Similar observations in those KTR were obtained for homoarginine and guanidinoacetate (Kayacelebi et al. 2017; Hanff et al. 2019a), two Arg metabolites formed by the catalytical action of arginine:glycine amidinotransferase (AGAT) (Tsikas and Wu 2015).

The aim of present study was to measure urinary concentrations of several AGEs, including CML, CEL and furosine, and their common precursor Lys, in urine samples of KTR and healthy donors, and to investigate potential associations between AGEs, transplantation outcome and donation of a kidney. To reach this goal, we newly analyzed urine samples for AGEs and amino acids by means of a validated gas chromatography–mass spectrometry (GC–MS) method that uses deuterium-labeled AGEs as internal standards (Baskal et al. 2021a).

## Materials and methods

### Design and study population

For the current study, we used material from a prospective cohort study among stable KTR in Northern Netherlands (TransplantLines Food and Nutrition Biobank and Cohort Study, Clinicaltrials.gov No NCT02811835), that has been described in detail previously (van den Berg et al. 2012, 2013, 2014). In this study, a total of 705 KTR and 321 living kidney donors participated. All subjects gave their written informed consent and all patients were transplanted at the University Medical Center Groningen (UMCG). The main inclusion criterion was having a renal graft that had been functioning for at least one year. Main exclusion criteria were drug and alcohol abuse, overt congestive heart failure (NYHA 3–4), malignancy (other than cured skin cancer) and an insufficient understanding of the Dutch language. The baseline examination of each participant was performed between November 2008 and March 2011, and participants were followed up until the end of August 2015. The data of 630 KTR were included for statistical analyses. In addition, a convenience sample of 41 healthy kidney donors (51% females; mean age, 53 years) derived from the same cohort study had both pre- and post-donation data available, and were, therefore, included in the study. The study protocol (METc 2008/186) was approved by the institutional ethical review board of the UMCG and has been conducted in accordance with the declaration of Helsinki. The primary outcome measure of the study was all-cause mortality. Secondary endpoints were cardiovascular mortality and death-censored graft loss (defined as return to dialysis or re-transplantation). The continuous surveillance system of the outpatient program ensured up-to-date information on patient status. Endpoints were recorded until September 2015 by a qualified physician. There was no loss that was due to follow-up for the primary endpoints.

### Clinical measurements

Each participant received written and verbal instructions on how to collect 24 h urine on the day prior to the visit to the outpatient clinic. Instructions were to start in the evening 2 days before the visit with emptying of the bladder in the toilet and writing down of the time of the voiding. After that, every urinary voiding would be added to a container, which was kept in the refrigerator at approximately 4 °C, until the same time in the evening was reached as the day before. At that time point, the bladder would be emptied again, and this urine would be added to the container, after which further urine collection was stopped. In the next morning, after an overnight fasting period, all participants (included KTR and healthy kidney donors) visited the outpatient clinic. Anthropometric measurements were performed on the same day as blood collection and urine collection was handed over. A strict protocol was followed for the measurements of blood pressure (mmHg) and heart rate with a semi-automatic device (Dinamap^®^ 1846, Critikon, Tampa, FL, USA) every minute for the duration of 15 min; the final value was defined as the average of the last three values. Detailed descriptions of anthropometric measurements have been described before (van den Berg et al. 2012, 2013, 2014). For routine clinical chemistry assays, heparin plasma was analyzed spectrophotometrically on the same morning using automated and validated routine methods (Roche Diagnostics, Basel, Switzerland).

### GC–MS measurement of urinary amino acids, their PTM metabolites and AGEs

The urine samples were transferred from UMCG frozen on dry ice to the Institute of Toxicology at Hannover Medical School and stored there (at − 20 °C) until analyses. Lys and AGEs were measured by GC–MS in 10-µL urine aliquots after solvent evaporation to dryness and a two-step derivatization first with methanolic 2 M HCl and then with pentafluoropropionic anhydride in ethyl acetate as described elsewhere in detail (Baskal et al. 2021a). This method is based on a GC–MS method previously reported for free amino acids in plasma and urine (Hanff et al. 2019b). GC–MS analyses were performed on the model ISQ from ThermoFisher (Dreieich, Germany) in the selected-ion monitoring mode (SIM) as reported in detail elsewhere (Baskal et al. 2021a).

The precision of the method in terms of relative standard deviation (RSD) ranged between 1.73% and 3.25%. The accuracy of the method in terms of recovery ranged between 109 and 120% for the AGEs added to a pooled quality control urine sample donated by a healthy volunteer. These data underline the high analytical reliability of the GC–MS method for the urinary AGEs measured in the present study.

### Statistical analyses

Data analyses and computations were performed with SPSS 24.0 software (IBM, Armonk, NY, USA), Stata SE version 15 (StataCorp, College Station, TX, USA), R version 3.5.1 software (The R-Foundation for Statistical Computing), and GraphPad Prism version 5 (GraphPad Software).

Baseline data are presented as means ± standard deviation for normally distributed data, as medians [interquartile range, IQR] for non-normally distributed data, and as numbers (percentages) for nominal data. A two-sided *P* value < 0.05 was considered to indicate statistical significance. In our initial analyses, we aimed to investigate pre- to post-donation changes in urinary excretion rates of Lys, CML and furosine, using Wilcoxon signed rank tests. Second, we aimed to compare the data of kidney donors to data of KTR, using Mann–Whitney *U* tests. Additionally, baseline characteristics are provided, along with the results of linear regression models adjusted for sex. Variables were log_2_ transformed if necessary to fulfill the assumptions for linear regression.

Prospective analyses of urinary AGEs excretion were performed for all-cause mortality, cardiovascular mortality and non-cardiovascular mortality. Prospective analyses were performed using uni- and multivariable Cox regression models. Adjustments were made for a priori selected variables, including age, sex, body mass index (BMI), estimated glomerular filtration rate (eGFR), proteinuria, cardiovascular risk factors and transplantation-related factors. Cardiovascular risk factors included total cholesterol, HDL cholesterol, systolic blood pressure, antihypertensive treatment, smoking (current, ex, or never) and type 2 diabetes. Transplantation-related factors were defined as donor type (deceased versus living), dialysis vintage, time between transplantation and baseline, cold ischemia time, calcineurin inhibitor usage, proliferation inhibitor usage, and the number of transplantations up to baseline. Causal pathway analyses were performed in which we adjusted for sodium, potassium, urea, and creatinine excretion. The proportionality of hazards assumption was tested with the Schoenfeld residual test, and was not violated for the associations of urinary AGEs excretion with all-cause mortality, cardiovascular mortality and non-cardiovascular mortality (*P* > 0.05 for all). Potential interactions for covariates were assessed by calculating interaction term, *P *interaction < 0.05 was considered to indicate significant effect-modification. To visualize the continuous associations of urinary AGEs with all-cause mortality and cardiovascular mortality, urinary AGEs excretion rates were plotted against the risk of all-cause mortality and cardiovascular mortality, respectively.

## Results

### Urinary excretion rates of amino acids, their PTM metabolites and AGEs in KTR and healthy kidney donors before and after donation

The urinary excretion rates (µmol/24 h) of amino acids, selected free PTM metabolites, and free AGEs in the healthy donors pre- and post-donation of a kidney and in KTR are summarized in Table S1. This table also summarizes the percentage changes as the result of the kidney donation by the 41 healthy donors. For a comparison, Table S1 also lists the molar ratios calculated by dividing the median excretion rates of KTR by those of the donors pre-donation (Pre) and post-donation (Post): KTR/Pre, KTR/Post. The molar ratios Post/Pre were calculated to visualize the effect of kidney donation in the donors.

Mean measured glomerular filtration rate (mGFR) was 120 ± 25 mL/min/1.73 m^2^ before donation, and 76 ± 14 mL/min/1.73 m^2^ after donation, indicating a considerable drop (− 37%) in the glomerular filtration rate. With the sole exception of CEA (+ 11%), the excretion rates of the other solutes decreased, varying from − 24% for CEL to − 62% for ADMA. The mean decrease was -45 ± 11% when considering all amino acids. Lys excretion rate dropped from 117 [81–203] µmol/24 h before donation to 51.3 [41–88] µmol/24 h after donation (− 56%). CML excretion rate was 10.6 [6.1–18] µmol/24 h before donation and 7.8 [4.9–11.9] µmol/24 h after donation (− 26%). Furosine excretion rate was 1.63 [1.21–2.44] µmol/24 h before donation and 0.89 [0.47–1.32] µmol/24 h after donation (− 45%). The decreases in the excretion rates were all significant (*P* < 0.01 for all solutes).

For a comparison, Table S1 also lists the molar ratios calculated by dividing the median excretion rates in KTR by the median excretion rates in donor post-donation (Post). The urinary excretion rates of amino acids and their metabolites were measured in 630 KTR. Their mean age was 53 ± 13 years, 263 (41.7%) of the patients were female, the median time after transplantation was 5.1 [1.6–10.9] years, and the mean eGFR was 45 ± 19 mL/min/1.73 m^2^, i.e., lower than in the healthy donors after kidney donation. With exception of sarcosine (i.e., Sarc, methylglycine), CEA and CEC, the KTR/Pre ratio was lower than 1.0 (Table S1). With the sole exception of guanidino acetate (GAA), the KTR/Post ratio was above 1.0. The Post/Pre ratio was lower than 1.0, except for CEA which was 1.11. These changes indicate clear effects of kidney donation on amino acid excretion in the healthy donors.

The baseline characteristics of the KTR cohort are presented in Table 1 (see also Table S2). Median urinary excretion rates were 84 [55–131] μmol/24 h for Lys, 9.2 [6.0–12.2] μmol/24 h for CML, and 0.90 [0.62–1.28] μmol/24 h for furosine. These values are significantly lower when compared to the healthy kidney donors before donation (*P* < 0.01 for all). Lys, CML and furosine excretion rates differed significantly between males and females, with lower rates in the latter (*P* < 0.01 for all).

**Table 1:** KTR characteristics at baseline and linear regression analyses for 24-h lysine, CML and furosine urinary excretion rates

	*n* = 630	Linear regression analyses, adjusted for sex					
Lysine excretion (µmol/24 h)	84 [55–131]	Lysine as dependent variable^a^		CML as dependent variable^a^		Furosine as dependent variable^a^	
CML excretion (µmol/24 h)	9.2 [6.1–12.2]						
Furosine excretion (µmol/24 h)	0.9 [0.6–1.3]	St. β (95% CI)	*P* value	St. β (95% CI)	*P* value	St. β (95% CI)	*P* value
Clinical characteristics							
Female sex, *n* (%)^b^	263 (42)	**− 0.45 (− 0.60 to 0.29)**	**< 0.001**^**c**^	**− 0.49 (− 0.64 to − 0.33)**	**< 0.001**	**− 0.41 (− 0.57 to − 0.26)**	**< 0.001**
Age, y	53 (13)	**− 0.11 (− 0.18 to − 0.03)**	**0.006**	− 0.02 (− 0.10 to 0.06)	0.6	− **0.08 (**− **0.16 to **− **0.00)**	**0.042**
Primary renal disease, *n* (%)							
Unknown	93 (15)	Ref		Ref		Ref	
Glomerulonephritis	162 (26)	0.03 (**− **0.22 to 0.28)	0.8	0.07 (**− **0.18 to 0.32)	0.6	0.06 (**− **0.20 to 0.30)	0.7
Interstitial nephritis	80 (13)	0.17 (**− **0.12 to 0.47)	0.2	0.09 (**− **0.20 to 0.39)	0.6	0.11 (**− **0.19 to 0.40)	0.5
Cystic kidney disease	131 (21)	**− **0.04 (**− **0.30 to 0.22)	0.8	0.03 (**− **0.23 to 0.29)	0.8	**− **0.09 (**− **0.36 to 0.17)	0.5
Other congenital/hereditary disease	34 (5)	**− **0.04 (**− **0.43 to 0.34)	0.8	**− **0.10 (**− **0.48 to 0.28)	0.6	**− **0.07 (**− **0.45 to 0.32)	0.7
Renal vascular disease	36 (6)	0.37 (**− **0.00 to 0.75)	0.053	0.26 (**− **0.12 to 0.63)	0.2	0.06 (**− **0.32 to 0.43)	0.8
Diabetic nephropathy	33 (5)	**− **0.01 (**− **0.39 to 0.38)	1.0	**− **0.11 (**− **0.50 to 0.28)	0.6	**− 0.46 (− 0.84 to − 0.07)**	**0.021**
Other multisystem diseases	44 (7)	0.09 (**− **0.26 to 0.44)	0.6	0.25 (**− **0.10 to 0.60)	0.2	0.25 (**− **0.10 to 0.61)	0.2
Other	17 (3)	0.33 (**− **0.18 to 0.83)	0.2	0.29 (**− **0.22 to 0.79)	0.3	0.25 (**− **0.25 to 0.76)	0.3
Height, cm	174 (10)	0.03 (**− **0.08 to 0.13)	0.6	0.01 (− 0.09 to 0.11)	0.8	− 0.02 (− 0.12 to 0.08)	0.7
Weight, kg	81 (17)	**0.16 (0.08–0.24)**	**< 0.001**	**0.10 (0.02–0.18)**	**0.011**	0.06 (− 0.02 to 0.14)	0.2
Body surface area, m^2^	1.95 (0.22)	**0.16 (0.07–0.25)**	**< 0.001**	**0.10 (0.02–0.19)**	**0.021**	0.05 (− 0.03 to 0.14)	0.2
Body mass index, kg/m^2^	26.7 (4.9)	**0.15 (0.08–0.23)**	**< 0.001**	**0.10 (0.03–0.18)**	**0.007**	0.07 (− 0.01 to 0.14)	0.093
Systolic blood pressure, mmHg	136 (17)	0.06 (− 0.02 to 0.13)	0.1	0.03 (− 0.05 to 0.10)	0.5	− 0.03 (− 0.11 to 0.05)	0.4
Diabetes, *n* (%)	152 (24)	0.10 (− 0.08 to 0.28)	0.3	0.17 (− 0.01 to 0.35)	0.058	0.05 (− 0.13 to 0.23)	0.6
History of cardiovascular disease, *n* (%)	160 (25)	− 0.01 (− 0.19 to 0.17)	0.9	0.10 (− 0.08 to 0.26)	0.3	− 0.05 (− 0.23 to 0.13)	0.6
Smoking status, *n* (%)							
Never	237 (40)	Ref		Ref		Ref	
History of smoking	280 (48)	− 0.03 (− 0.20 to 0.14)	0.7	0.01 (− 0.16 to 0.18)	0.9	0.06 (− 0.11 to 0.23)	0.5
Current smoking	73 (12)	− 0.14 (− 0.40 to 0.12)	0.3	− 0.05 (− 0.31 to 0.21)	0.7	− 0.05 (− 0.31 to 0.21)	0.7
Pre-emptive transplantation, *n* (%)	104 (17)	0.05 (− 0.16 to 0.26)	0.6	− 0.08 (− 0.29 to 0.12)	0.4	0.03 (− 0.18 to 0.24)	0.8
Duration of dialysis, months^a^	24 [4–48]	− 0.04 (− 0.12 to 0.03)	0.3	0.01 (− 0.07 to 0.08)	0.9	− 0.03 (− 0.10 to 0.05)	0.5
Time after transplantation, y^a^	5.1 [1.6–10.9]	− 0.05 (− 0.13 to 0.03)	0.2	− 0.02 (− 0.10 to 0.06)	0.6	− 0.06 (− 0.13 to 0.02)	0.15
History of rejection, *n* (%)	161 (26)	− 0.10 (− 0.28 to 0.08)	0.3	− 0.11 (− 0.29 to 0.06)	0.2	− 0.11 (− 0.28 to 0.07)	0.2
History of delayed graft function, *n* (%)	48 (8)	0.04 (− 0.25 to 0.33)	0.8	0.07 (− 0.22 to 0.36)	0.6	0.10 (− 0.19 to 0.38)	0.5
Anti-HLA Class II antibodies, *n* (%)	106 (17)	− 0.09 (− 0.30 to 0.12)	0.4	− 0.15 (− 0.35 to 0.06)	0.2	− 0.13 (− 0.34 to 0.08)	0.2
Donor age, y	43 (15)	− 0.04 (− 0.12 to 0.04)	0.3	0.01 (− 0.07 to 0.08)	0.9	− 0.03 (− 0.10 to 0.05)	0.5
Living donor, *n* (%)	221 (35)	0.05 (− 0.11 to 0.21)	0.5	− 0.08 (− 0.24 to 0.08)	0.3	0.11 (− 0.05 to 0.27)	0.2
Cold ischemia time, h^a^	15 [2.8–21]	− **0.09 (**− **0.17 to **− **0.014)**	**0.021**	− 0.01 (− 0.09 to 0.06)	0.7	− **0.11 (**− **0.18 to **− **0.03)**	**0.007**
Laboratory measurements							
Hemoglobin, g/dL	8.2 (1.1)	**0.16 (0.09–0.24)**	**< 0.001**	**0.18 (0.11–0.26)**	**< 0.001**	**0.22 (0.14–0.30)**	**< 0.001**
Sodium mmol/L	141 (2.8)	**0.13 (0.05–0.20)**	**0.001**	**0.20 (0.14–0.29)**	**< 0.001**	**0.17 (0.09–0.24)**	**< 0.001**
Potassium, mmol/L	4.0 (0.5)	**− 0.11 (− 0.19 to − 0.03)**	**0.007**	**− **0.05 (**− **0.13 to 0.03)	0.2	**− **0.07 (**− **0.15 to 0.01)	0.072
Creatinine, µmol/L^a^	125 [100–160]	**− 0.13 (− 0.21 to − 0.05)**	**0.001**	**− 0.13 (− 0.21 to 0.05)**	**0.002**	**− 0.30 (− 0.37 to − 0.22)**	**< 0.001**
Cystatin C, mg/L	1.85 (0.79)	**− 0.15 (− 0.23 to − 0.07)**	**< 0.001**	**− 0.11 (− 0.19 to − 0.03)**	**0.004**	**− 0.32 (− 0.39 to − 0.24)**	**< 0.001**
eGFR, mL/min/1.73 m^2^	45 (19)	**0.21 (0.13–0.29)**	**< 0.001**	**0.13 (0.05–0.20)**	**0.001**	**0.32 (0.24–0.39)**	**< 0.001**
Urea, mmol/L^a^	9.5 [7.2–13.3]	**− 0.19 (− 0.26 to − 0.11)**	**< 0.001**	− **0.10 (**− **0.17 to **− **0.02)**	**0.012**	− **0.27 (**− **0.34 to **− **0.19)**	**< 0.001**
Triglycerides, mmol/L^a^	1.7 [1.3–2.3]	0.02 (**− **0.06 to 0.10)	0.6	0.04 (− 0.04 to 0.11)	0.4	− 0.01 (− 0.09 to 0.07)	0.9
HDL cholesterol, mmol/L^a^	1.3 [1.1–1.6]	**− **0.04 (**− **0.13 to 0.04)	0.3	− 0.03 (− 0.11 to 0.06)	0.5	0.05 (− 0.03 to 0.13)	0.2
LDL cholesterol, mmol/L	2.95 (0.92)	0.02 (− 0.06 to 0.10)	0.6	0.03 (− 0.05 to 0.11)	0.4	0.04 (− 0.04 to 0.11)	0.4
HbA1c, %^a^	5.8 [5.5–6.2]	0.07 (− 0.01 to 0.15)	0.082	0.13 (0.06–0.21)	**0.001**	0.08 (0.01–0.16)	**0.035**
Leukocyte count, 10^9^/L	8.1 (2.6)	0.03 (− 0.05 to 0.11)	0.4	0.08 (0.00–0.16)	**0.038**	0.03 (− 0.05 to 0.11)	0.5
hs-CRP, mg/L^a^	1.6 [0.7–4.6]	0.01 (− 0.07 to 0.09)	0.7	0.02 (− 0.06 to 0.10)	0.6	− 0.07 (− 0.14 to 0.01)	0.097
Albumin, g/L	43.0 (3.0)	0.05 (− 0.03 to 0.12)	0.3	0.03 (− 0.05 to 0.11)	0.4	0.12 (0.04–0.20)	**0.003**
Urinary protein excretion, g/24 h	0.40 (0.81)	0.07 (− 0.01 to 0.14)	0.091	− 0.08 (− 0.16 to − 0.00)	**0.042**	− 0.12 (− 0.20 to − 0.04)	**0.002**
Urinary sodium excretion, mmol/24 h	158 (63)	0.28 (0.20–0.36)	**< 0.001**	0.25 (0.18–0.33)	**< 0.001**	0.26 (0.18–0.33)	**< 0.001**
Urinary urea excretion, mmol/24 h	392 (114)	0.25 (0.17–0.33)	**< 0.001**	0.22 (0.14–0.30)	**< 0.001**	0.29 (0.21–0.37)	**< 0.001**
Urinary creatinine excretion, mmol/24 h	11.8 (3.5)	0.27 (0.18–0.36)	**< 0.001**	0.22 (0.13–0.31)	**< 0.001**	0.30 (0.21–0.39)	**< 0.001**
Medication							
Angiotensin-2 antagonist, *n* (%)	95 (15)	− 0.11 (− 0.32 to 0.11)	0.3	− 0.03 (− 0.24 to 0.19)	0.8	− 0.09 (− 0.31 to 0.13)	0.4
ACE-inhibitor, *n* (%)	200 (32)	− 0.04 (− 0.21 to 0.12)	0.6	− 0.03 (− 0.19 to 0.14)	0.8	− 0.01 (− 0.18 to 0.16)	0.9
Betablocker, *n* (%)	403 (64)	− 0.09 (− 0.25 to 0.07)	0.3	− 0.08 (− 0.24 to 0.08)	0.3	− 0.02 (− 0.18 to 0.13)	0.8
Calcium antagonist, *n* (%)	154 (24)	0.27 (0.10–0.45)	**0.002**	0.03 (− 0.15 to 0.21)	0.8	− 0.18 (− 0.34 to − 0.02)	**0.032**
Diuretic, *n* (%)	250 (40)	− 0.26 (− 0.41 to − 0.10)	**0.001**	0.00 (− 0.15 to 0.16)	1.0	− 0.05 (− 0.23 to 0.13)	0.6
Prednisolone, *n* (%)	627 (100)	− 0.36 (− 1.47 to 0.75)	0.5	**1.77 (0.68–2.87)**	**0.002**	0.33 (− 0.78 to 1.45)	0.6
Calcineurin inhibitor, *n* (%)	359 (57)	− 0.06 (− 0.21 to 0.10)	0.5	− 0.03 (− 0.19 to 0.12)	0.7	− 0.08 (− 0.24 to 0.07)	0.3
Proliferation inhibitor, *n* (%)	537 (85)	0.04 (− 0.18 to 0.25)	0.7	− 0.11 (− 0.33 to 0.10)	0.3	0.01 (− 0.21 to 0.22)	1.0
mTOR inhibitor, *n* (%)	20 (3)	0.41 (− 0.03 to 0.84)	0.067	0.15 (− 0.28 to 0.59)	0.5	0.21 (− 0.23 to 0.64)	0.4

Normally distributed data are presented as mean ± standard deviation, skewed data as median [interquartile range], and categorical data as number (valid percentage)

^a^Variables were log_2_ transformed to fulfill the assumptions in linear regression analyses

^b^Linear regression results are derived from a univariable model for sex. Diabetes was defined according to the American Diabetes Association criteria. Data on smoking status was missing in 45 patients (7.1%), data on donor age was missing in 16 patients (2.5%), data on HbA1c were missing in 23 patients (3.7%), and data on hs-CRP were missing in 35 patients (5.6%). All other variables had missing data for ≤ 10 patients. *eGFR* estimated glomerular filtration rate as calculated using the creatinine and cystatin C-based CKD-EPI formula, *hs-CRP* high-sensitivity C-reactive protein

^**c**^Numbers in bold indicate statistical significance

Linear regression analyses adjusted for sex are presented in Table 1. Notably, body weight was an important determinant of Lys (St. β 0.16; 95% CI 0.08–0.24, *P* < 0.001), and to a lesser extent of CML (St. β 0.10; 95% CI 0.02–0.18, *P* = 0.011), but not of furosine, and was independent of sex. Additionally, parameters associated with worse kidney function, including cold ischemia time, creatinine, cystatin C and urea, were all associated with lower Lys, CML and furosine excretion rates, and were independent of sex. In contrast, the associations of Lys, CML and furosine with eGFR and hemoglobin were positive (*P* < 0.001 for all). In addition, urinary sodium, urea and creatinine excretions were positively associated with Lys, CML and furosine excretion rates.

The urinary excretion of furosine was inversely associated with the intake of antihypertensive drugs (St. β − 0.34, *P* = 0.005). To our knowledge, there is no report in the literature on such an association in health and disease. Calcium antagonist use was associated inversely with furosine and positively with Lys excretion rates, while diuretics use was strongly inversely associated with Lys. It is also interesting to note, that furosine excretion rate was inversely associated with nephropathy, while the excretion rate of CML was associated with prednisolone, which is a widely used immunosuppressive in organ transplantations.

### Prospective analyses

**Fig. 1:**
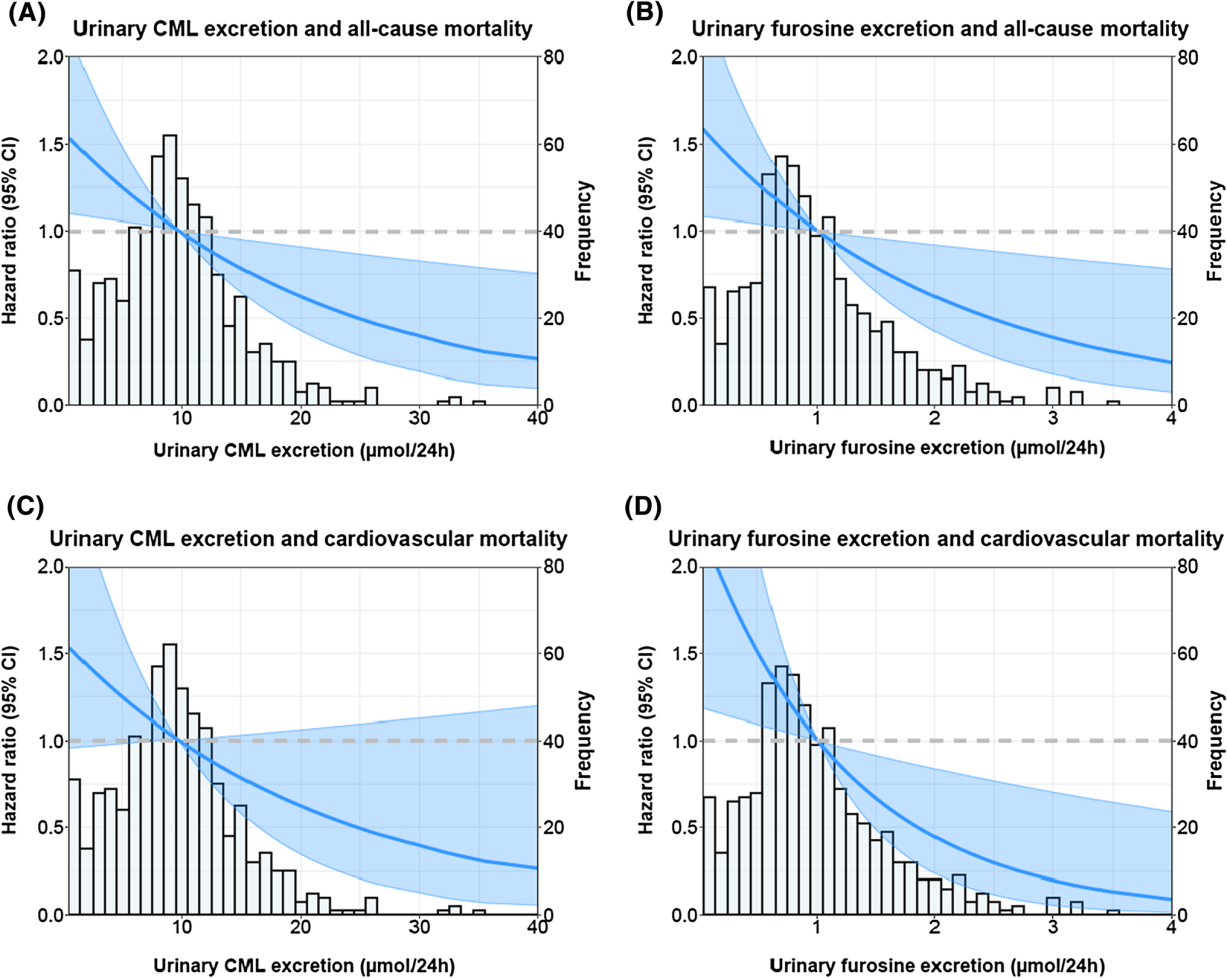
Associations of urinary excretion rates of **A** CML and **B** furosine with all-cause mortality in the KTR, and of **C** CML and **D** furosine with cardiovascular mortality. The lines show the adjusted hazard ratio (HR) and the shaded area corresponds to the 95% pointwise confidence interval (CI). The analyses were adjusted for age, sex, BMI, eGFR and proteinuria

During a median follow-up of 5.3 [4.7–6.0] years, 135 (21%) KTR died, of which 56 (41%) died due to cardiovascular causes, and 79 (59%) due to non-cardiovascular causes. Univariable and multivariable Cox regression analyses of urinary Lys, CML and furosine excretion with all-cause mortality, cardiovascular mortality and non-cardiovascular mortality are summarized in Tables 2, 3 and in Fig. 1.

**Table 2:** Association of urinary excretion rates of Lys, CML and furosine with all-cause mortality, cardiovascular mortality, and non-cardiovascular mortality in KTR

	All-cause mortality		Cardiovascular mortality		Non-cardiovascular mortality	
	HR per SD increase [95% CI]	*P*	HR per SD increase [95% CI]	*P*	HR per SD increase [95% CI]	*P*
Lysine						
Model 1	0.79 [0.59–1.07]	0.14	0.92 [0.66–1.28]	0.62	0.68 [0.42–1.10]	0.11
Model 2	0.83 [0.62–1.10]	0.20	0.90 [0.63–1.27]	0.54	0.76 [0.48–1.19]	0.23
Model 3	0.87 [0.67–1.12]	0.28	0.91 [0.65–1.27]	0.57	0.83 [0.56–1.23]	0.35
Model 4	0.87 [0.64–1.18]	0.37	0.88 [0.56–1.40]	0.59	0.87 [0.58–1.29]	0.48
Model 5	0.90 [0.70–1.15]	0.40	0.94 [0.68–1.31]	0.72	0.86 [0.59–1.25]	0.43
CML						
Model 1	0.77 [0.64–0.94]	0.009	0.82 [0.62–1.10]	0.19	0.74 [0.57–0.95]	0.02
Model 2	0.74 [0.61–0.91]	0.004	0.75 [0.56–1.01]	0.06	0.73 [0.56–0.96]	0.03
Model 3	0.78 [0.64–0.96]	0.02	0.76 [0.56–1.04]	0.09	0.80 [0.61–1.06]	0.11
Model 4	0.72 [0.57–0.90]	0.004	0.69 [0.49–0.97]	0.03	0.75 [0.56–1.01]	0.05
Model 5	0.79 [0.64–0.97]	0.02	0.74 [0.54–1.02]	0.07	0.81 [0.61–1.08]	0.15
Furosine						
Model 1	0.61 [0.48–0.76]	< 0.001	0.54 [0.38–0.78]	< 0.001	0.66 [0.50–0.87]	0.003
Model 2	0.62 [0.49–0.78]	< 0.001	0.53 [0.36–0.77]	< 0.001	0.70 [0.52–0.93]	0.02
Model 3	0.75 [0.59–0.96]	0.03	0.59 [0.39–0.90]	0.01	0.89 [0.65–1.21]	0.44
Model 4	0.74 [0.57–0.97]	0.03	0.57 [0.37–0.89]	0.01	0.89 [0.65–1.22]	0.47
Model 5	0.78 [0.60–0.99]	0.05	0.60 [0.39–0.91]	0.02	0.94 [0.69–1.28]	0.69

Model 1: Crude model

Model 2: Model 1 + Age, sex and BMI

Model 3: Model 2 + eGFR and proteinuria

Model 4: Model 3 + cardiovascular risk factors (total cholesterol, HDL cholesterol, systolic blood pressure, antihypertensive treatment, smoking (current, ex, or never] and diabetes]

Model 5: Model 3 + transplantation related factors (donor type, total dialysis time, time from transplantation to baseline, cold ischemia time, CNI usage, proliferation inhibitor usage and transplantation count]

eGFR was calculated according to the Chronic Kidney Disease Epidemiology formula with plasma creatinine and plasma cystatin C

Proportional hazards assumption was not violated in any of the models

**Table 3:** Association of urinary excretion rates of lysine, CML and furosine with graft failure

	Graft failure	
	HR per SD increase [95% CI]	*P* value
Lysine		
Model 1	0.92 [0.69–1.21]	0.54
Model 2	0.87 [0.64–1.19]	0.39
Model 3	0.89 [0.68–1.16]	0.40
Model 4	0.93 [0.71–1.22]	0.61
Model 5	0.89 [0.68–1.16]	0.39
CML		
Model 1	0.84 [0.65–1.07]	0.16
Model 2	0.79 [0.61–1.02]	0.07
Model 3	0.95 [0.74–1.23]	0.71
Model 4	0.99 [0.78–1.29]	0.99
Model 5	0.96 [0.74–1.24]	0.74
Furosine		
Model 1	0.50 [0.36–0.68]	< 0.001
Model 2	0.44 [0.32–0.61]	< 0.001
Model 3	0.76 [0.54–1.08]	0.13
Model 4	0.80 [0.57–1.13]	0.21
Model 5	0.78 [0.55–1.11]	0.16

Model 1: Crude model

Model 2: Model 1 + Age, sex and BMI

Model 3: Model 2 + eGFR^a^ and proteinuria

Model 4: Model 3 + cardiovascular risk factors (total cholesterol, HDL cholesterol, systolic blood pressure, antihypertensive treatment, smoking [current, ex, or never] and diabetes)

Model 5: Model 3 + transplantation related factors (donor type, total dialysis time, time from transplantation to baseline, cold ischemia time, CNI usage, proliferation inhibitor usage and transplantation count)

^a^eGFR was calculated according to the Chronic Kidney Disease Epidemiology formula with plasma creatinine and plasma cystatin C

Proportional hazards assumption was not violated in any of the models

No association was found between urinary Lys excretion rate and all-cause mortality, cardiovascular mortality or non-cardiovascular mortality. Urinary CML excretion rate was inversely associated with all-cause mortality (Hazard Ratio (HR) per SD increase: 0.77 [0.64–0.94]; *P* = 0.009) and non-cardiovascular mortality (HR: 0.74 [0.57–0.95]; *P* = 0.02). The association of urinary CML excretion rate with all-cause mortality remained significant after adjustment for potential confounders, while the association with non-cardiovascular mortality was lost after adjusting for transplantation-related factors. Urinary furosine excretion rate was inversely associated with all-cause mortality (HR: 0.61 [0.48–0.76]; *P* < 0.001) (Fig. 1), cardiovascular mortality (HR: 0.54 [0.38–0.78]; *P* < 0.001) and non-cardiovascular mortality (HR: 0.66 [0.50–0.87]; *P* = 0.003). The associations of urinary furosine excretion with all-cause mortality and cardiovascular mortality remained significant after adjustment for potential confounders, while the association with non-cardiovascular mortality was lost after adjusting for eGFR and proteinuria.

Associations of urinary CML and furosine excretion rates with all-cause mortality and cardiovascular mortality in KTR are shown in Fig. 1. Causal pathway analyses demonstrated that the associations of urinary CML and furosine excretion rates with all-cause mortality were lost after adjustment for urinary urea and creatinine excretion, reflecting protein intake and muscle mass, respectively (Table S3).

## Discussion

The kidney is a multifunctional organ, with functions depending on and independent of the nephron. About 20% of the blood volume that enters the kidneys is filtered through the glomerulus. The estimated glomerulus filtration rate (eGFR) is a measure of kidney function. Substances needed by the body (e.g., water, electrolytes, amino acids, glucose) are reabsorbed from the ultrafiltrate by transporters specific for charge-free and charged substances. In kidney failure, eGFR decreases below 15% and necessitates dialysis and kidney transplantation. Donation of a kidney by a healthy subject is not only a life-saving measure for the patient, but it also concerns the donor’s post-donation life as it forces them to live with a single kidney. Beyond these crucially important issues, kidney transplantation is a unique clinical condition to study various aspects of kidney’s physiology and pathology. Furthermore, studies aiming to improve kidney transplantation by prolonging survival and improving life post-transplantation are warranted.

Previously, in a cohort of 555 stable KTR (mean eGFR, 47 mL/min/1.73 m^2^), CML and CEL, two AGEs of Lys, were measured by LC–MS/MS in plasma at mean concentrations of 374 ng/mL (1.83 µM) and 224 ng/mL (1.03 µM), respectively, with a mean CML-to-CEL molar ratio of 1.8. Circulating CML and CEL were found to be independently associated with long-term risk of cardiovascular mortality (Sotomayor et al. 2019). In the present study, we measured by GC–MS in 24 h collected urine samples of another cohort of 630 KTR (mean eGFR, 45 mL/min/1.73 m^2^) and in 41 healthy donors, the excretion of a wide spectrum of free amino acids, and many of the free PTM metabolites and of the free AGEs of Lys, Arg and Cys. The focus of the present study was on Lys and its AGEs, notably CML, CEL and furosine. In the donors, the median excretion rates were (pro-donation/post-donation) 10.6/7.8 µmol/24 h for CML, 8.06/6.15 µmol/24 h for CEL and 1.63/0.89 µmol/24 h for furosine. The CML-to-CEL molar ratio was 1.32 pre- and 1.27 post-donation. In the KTR, the median excretion rates of CML (9.2 µmol/24 h) and CEL (6.86 µmol/24 h) were lower compared to those of the donors pre-donation, but higher compared to post-donation. The CML-to-CEL molar ratio was 1.34, i.e., very close to that of the donors.

Kidney donation resulted in a decrease of the eGFR by − 37%. With the sole exception of CEA, an AGE of Arg, the urinary excretion rates of all other AGEs, PTM metabolites and native amino acids decreased in the range − 24% to − 62% (mean − 45%). This decrease is of the order of the drop in eGFR in the healthy donors. The diverging effect of CEA (+ 11%) is strange, especially when comparing with CMA (− 29%), an AGE of Arg as well. Methylglyoxal (MGO) and glyoxal (GO) are the precursors of CEA and CMA, respectively. Presumably, kidney donation may have induced changes in the synthesis of MGO and GO. The latter compounds are excreted in the urine of healthy humans at mean concentrations each of about 500 nM (Ojeda et al. 2014). In serum of healthy humans, the mean MGO and GO concentrations were reported to be about 98 and 150 nM, respectively (Dhananjayan et al. 2019).

In the previous KTR cohort (Sotomayor et al. 2019), higher plasma concentrations of CML and CEL were associated with higher risk for cardiovascular and all-cause mortality. In the KTR of the present study, urinary CML and furosine showed the strongest associations: lower CML and lower furosine excretion rates were found to be associated with higher all-cause mortality. In addition, the urinary excretion of furosine was associated with cardiovascular mortality. Such associations were not found for Lys, the common precursor of CML and furosine. In the same KTR cohort, we found that higher plasma concentrations of ADMA, a major PTM metabolite of Arg, were associated with a higher risk for cardiovascular and all-cause mortality (Frenay et al. 2015a), whereas higher urinary excretions of ADMA and its isomer SDMA were associated with a lower risk for mortality (Said et al. 2019b). By contrast, lower plasma concentrations of homoarginine and higher excretion rates of homoarginine were found to be associated with higher mortality (Frenay et al. 2015b; Kayacelebi et al. 2017). Possibly, PTM metabolites (such as ADMA and SDMA) and AGEs (such as CML) are associated with mortality in KTR in a similar manner, in contrast to homoarginine, taurine and creatine (Post et al. 2019a, 2019b), presumably due to their further utility in the body.

The biological functions of non-histone proteins modified by post-translation modification and glycation are only little understood. Better understood are low molecular mass free PTM metabolites and the AGEs released by proteolysis from modified proteins. Thus, ADMA is known to inhibit the activity of nitric oxide synthase (NOS) isoforms that convert l-arginine to l-citrulline and nitric oxide (NO), one of the most potent endogenous vasodilators. SDMA is considered not to be an inhibitor of NOS activity, although it has been reported to inhibit the activity of neuronal NOS, albeit less strongly than ADMA (Tsikas et al. 2000). Despite some inconsistencies and unexplained observations, high ADMA and SDMA production and low homoarginine production are generally assumed to be exclusive NO-related causes for cardiovascular morbidity and mortality in diseases of various organs including the heart and the kidneys (Tsikas et al. 2018). Whether the free *N*^ε^-mono-, -di-, and -trimethylated Lys metabolites, i.e., MML, DML and TML, respectively, are involved in certain pathways at relevant concentration ranges analogous to ADMA is unknown. In plasma, high TML concentrations were found to predict major adverse cardiovascular events (MACE) in patients with acute or stable coronary artery disease (CAD), but not in acute ischemic stroke (Schwedhelm et al. 2021). In our study, urinary MML behaved similarly to ADMA both in the KTR and in the healthy donors. The biological activity of MML, DML and TML remains to be investigated.

AGEs are assumed to exert their biological activities via their receptor for advanced glycation end-products (RAGE), which exists in two forms, as membrane-bound (mRAGE) and soluble (sRAGE). CML- and CEL-containing synthetic heptapeptides were found to have very high binding affinity constants, which were about 8 times lower compared to the non-glycated peptides (87 µM vs. 673 µM) (Xue et al. 2011). This observation suggests that the carboxymethyl and carboxyethyl groups of CML and CEL increase the affinity of these AGEs to the V-domain of RAGE. AGEs derived from long-term in vitro glycation of bovine serum albumin (BSA-AGEs, MW 67.8–78.6 kDa) were found to have much higher affinity (about 1 µM) to soluble human RAGE, with glyoxylic acid-glycated BSA being the most affine (about 0.1 µM) (Valencia et al. 2004).

RAGE has been linked to several chronic diseases, which share that they are thought to result from alterations in the micro- and macrovasculature, together leading to organ damage and ultimately even to organ failure (Gasparotto et al. 2017, 2019). In KTR, AGEs have been hypothesized to play a role in the pathogenesis of cardiovascular disease, i.e., in the initiation and progression of cardiovascular disease. In our previous study, we found that circulating CML and CEL are prospectively associated with long-term risk of cardiovascular mortality in 555 stable KTR (Sotomayor et al. 2019). The present study comprising 630 stable KTR suggests that lower excretion rates of CML and furosine are associated with higher all-cause mortality. In addition, furosine was also associated with cardiovascular mortality. Although we did not measure circulating AGEs in these KTR, the combination of previous findings (Sotomayor et al. 2019), with the observations of the present study suggests that AGEs, notably of Lys (CML, CEL, furosine) and of Arg (CMA), accumulate in the blood because of their insufficient glomerular filtration in the failing kidney. In the mouse, furosine was reported to damage the kidney when administered chronically at very high doses (up to 0.5 g/kg), presumably by triggering ferroptosis, an iron-dependent apoptosis (Li et al. 2020).

In our study, urinary excretion of furosine, but not CML or Lys, was inversely associated with the intake of antihypertensive drugs (St. β − 0.34, *P* = 0.005). To our knowledge, there is no report in the literature on such an association in health and disease. Calcium antagonist use was associated inversely with furosine and positively with Lys excretion rates, while diuretics use was strongly inversely associated with Lys. As CML and Lys differ in their structures from furosine only in the furoyl residues (Scheme 1), it could be hypothesized that the furoyl residue of furosine is responsible for these associations. In a type 2 diabetic nephropathy rat model, two anti-hypertensive drugs of different mechanisms of action, i.e., olmesartan and hydralazine, were found to inhibit in vivo formation of protein pentosidine in the kidney and to improve renal damage (Nangaku et al. 2003). Analogous results were observed for various AGEs in vivo and in vitro for valsartan (Mil et al. 2021; see also Prasad and Mishra 2017).

It is also interesting to note, that furosine excretion rate was inversely associated with nephropathy, while the excretion rate of CML was associated with prednisolone which is a widely used immunosuppressive in organ transplantations. In our KTR cohort, 627 patients (100%) received prednisolone, which was positively associated with the CML excretion rate (St. β 1.77, *P* = 0.002). Methylprednisolone has been reported to induce the expression of *RAGE* genes in primary human keratinocytes (Djerbi et al. 2013). In the (NZB/NZW)F1 mice, intraperitoneal administration of sRAGE was found to alleviate nephritis, and it was as effective as the oral co-administration of mycophenolate and prednisolone (Lee et al. 2013). It has been hypothesized that in the extracellular space sRAGE binds to mRAGE, and by this way reduces the inflammation induced by NF-κB (Maillard-Lefebvre et al. 2009; Park et al. 2011).

Chemical synthesis of CML requires reductive condensation between Lys and glyoxalate and results in *N*,*N*-CML due to alkylation of both, the *N*^α^ and *N*^ε^ amine groups of Lys. The first enzymatic synthesis of CML has been reported in 1994 (Miller et al. 1994). This reaction was catalyzed by the NADPH-dependent enzyme *N*^5^-(carboxyethyl)ornithine synthase (EC 1.5.1.24), which is found in some strains of the lactic acid bacterium *Lactococcus lactis*.

In healthy Japanese subjects, a major fraction of about 90% of circulating free CML and CEL was found to be present in erythrocytes at mean concentrations of 0.9 µM and 0.2 µM (CML-to-CEL molar ratio, 4.5), respectively, as measured by LC–MS/MS (Nomi et al. 2020). The concentration of CML and CEL in blood was found to change after a meal (supplied with about 12.9 µmol CML and 3.3 µmol CEL), suggesting an endogenous source of erythrocytic CML and CEL. As aldehydes are the glycation components, it has been investigated whether the genes aldehyde dehydrogenase 2 (*ALDH2*) and 1B type alcohol dehydrogenase (*ADH1B*) are involved in the biosynthesis of CML and CEL with some evidence of a genetic polymorphism in CML metabolism (Nomi et al. 2020). We are not aware whether endogenous AGEs including CML and CEL are formed in humans enzymatically, chemically or both.

Strengths of the present study are the reliable non-invasive simultaneous measurement of free AGEs, PTM metabolites and amino acids in urine samples of the KTR and healthy kidney donors by GC–MS using stable-isotope labeled analogs. In our study, after each urination the container was put immediately into a refrigerator and kept at approximately 4 °C until the next urination. Under such collection and storage conditions, the AGEs measured in the present study are stable for at least 3 days in human and rat urine (Baskal et al. 2021a, 2021b). To the best of our knowledge, there is no need for stabilization of AGEs in urine or even in blood as demonstrated for CML and CEL (Hull et al. 2013). Obviously, AGEs are chemically and metabolically stable in urine and blood. Further strengths of our study are the large sample size of this well-defined cohort, the long follow-up and the collection of a wide variety of demographical and laboratory parameters allowing adjustment for many potential confounders. Nonetheless, the study limitations also need to be considered. Statistical significance in observational studies by nature does not confirm biological significance. It is unknown whether the relations between AGEs and PTM excretion rates and mortality are causal or associative. In addition, our study population consisted predominantly of Caucasian individuals, which precludes us from extrapolation of our results to populations of other ethnicities. Furthermore, the possibility of residual confounding remains a possibility. In our study, we did not measure the glycemic load of KTR and healthy donors. Yet, in previous large study, glycemic load was not associated with urinary CML or CEL (Maasen et al. 2019).

## Conclusion

Lower excretion rates of CML and furosine, but not of their common precursor Lys, are associated with higher all-cause mortality. In addition, lower excretion rate of furosine is associated with higher cardiovascular mortality in KTR. In the healthy donors of the study, kidney donation is associated with considerable decrease of the excretion rates of almost all amino acids, AGEs and PTM metabolites. The results of the present study suggest that renal excretion of modified and non-modified amino acids is altered in KTR compared to healthy kidney donors. The different associations of furosine with diabetic nephropathy and with the administration of anti-hypertensive drugs, and the association of CML with the intake of the immunosuppressant prednisolone warrants further in vitro and in vivo investigations. Next kidney transplantation studies should include measurements of sRAGE and mRAGE in donors and recipients.

## Supplementary Information

Below is the link to the electronic supplementary material.

Supplementary file1 (DOCX 41 KB)

## Abbreviations

AGEs: Advanced glycation end-products
AGE-BSA: AGEs of bovine serum albumin
BMI: Body mass index
BSA: Body surface area or bovine serum albumin
CAD: Coronary artery disease
CEA: *N*^G^-Carboxyethylarginine
CEC: *S*-(1-Carboxyethyl)-l-cysteine
CEL: *N*^6^-Carboxyethyl-l-lysine
CI: Confidence interval
CMC: *S*-Carboxymethyl-l-cysteine
CML: *N*^6^-Carboxymethyl-l-lysine
CVD: Cardiovascular disease
DML: *N*^6^-Dimethyl-l-lysine
GC–MS: Gas chromatography–mass spectrometry
GO: Glyoxal
HR: Hazard ratio
IQR: Interquartile range
KTR: Kidney transplant recipients
LC–MS/MS: Liquid chromatography–tandem mass spectrometry
MACE: Major adverse cardiovascular events
MGO: Methylglyoxal
MML: *N*^6^-Monomethyl-l-lysine
*m/z*: Mass-to-charge ratio
PKMT: Protein-lysine methyltransferase
PTM: Post-translational modification
RAGE: Receptor of AGEs
mRAGE: Membrane-bound RAGE
sRAGE: Soluble RAGE
TML: *N*^6^-Trimethyl-l-lysine
