# Supplementary material for: Urinary excretion of amino acids and their advanced glycation end-products (AGEs) in adult kidney transplant recipients with emphasis on lysine: furosine excretion is associated with cardiovascular and all-cause mortality
Source: Amino Acids. 2021 Oct 24;53(11):1679–93. doi: 10.1007/s00726-021-03091-8 (PMC8592953; doi:10.1007/s00726-021-03091-8)
Supplement: Supplementary file 1 — Supplementary file1 (DOCX 41 KB) [file 726_2021_3091_MOESM1_ESM.docx]

**Supplement to:**

**Urinary excretion of amino acids and their advanced glycation end-products (AGEs) in adult kidney transplant recipients with emphasis on lysine: Furosine excretion is associated with cardiovascular and all-cause mortality**

Svetlana Baskal^1^, Adrian Post^2^, Daan Kremer^2^, Alexander Bollenbach^1^, Stephan J.L. Bakker^2^, Dimitrios Tsikas^1,*^

^1^ Core Unit Proteomics, Institute of Toxicology, Hannover Medical School, 30623 Hannover, Germany

^2^ Division of Nephrology, Department of Internal Medicine, University Medical Center Groningen and University of Groningen, Groningen, The Netherlands

**Table S1**. Excretion rates (µmol/24 h) of amino acids and their PTM metabolites and AGEs in the urine by healthy humans pre- and post-donation of a kidney and by kidney transplant recipients (KTR)

____________________________________________________________________________________________________________________________________________

Amino acid Pre-donation (*n*=41) Post-donation (*n*=41) Change (%) KTR (*n*=632) KTR/Pre KTR/Post Post/Pre

____________________________________________________________________________________________________________________________________________

Ala 283 [171-421] 133 [95.7 – 197.5] -53 192 [123-332.8] 0.68 1.44 0.47

Thr 238 [149-341] 105 [67.6-156] -56 135 [88.7-106.8] 0.57 1.29 0.44

Gly 1766 [1328-2779] 768 [508.5-1363] -57 875.5 [547.8-1372] 0.50 1.14 0.43

Sarc 0.68 [0.505-1.07] 0.37 [0.265-0.535] -46 0.715 [0.47-1.08] 1.05 1.93 0.54

Val 63.8 [41.2-81.9] 30.9 [21.3-46.5] -52 38.0 [26.9-56.1] 0.59 1.23 0.48

Ser 518 [296.5-618 231 [148.5-363.5] -55 281.5 [190.3-407] 0.54 1.22 0.45

Leu/Ile 76.3 [53.8-104] 40.8 [26.6-55.9] -47 54.8 [36.7-81.5] 0.72 1.34 0.53

Asn/Asp 337 [192.5-442.5] 153 [123.5-221.5] -55 221 [152.3-319] 0.66 1.44 0.45

Pro 3.00 [2.09-4.31] 1.80 [1.33-4.50] -40 2.17 [1.50-3.23] 0.72 1.21 0.60

OH-Pro 0.97 [0.755-1.835] 0.69 [0.535-1.14] -29 0.94 [0.56-1.63] 0.97 1.36 0.71

Met 203 [151-263] 119 [95.1-166] -41 157.5 [120-199.5] 0.78 1.32 0.59

Gln/Glu 1486 [1119-2083] 774 [517.5-978.5] -48 1065 [783.3-1509] 0.72 1.38 0.52

Orn/Cit 44.3 [33.8-62.8] 26 [19.8-38.5] -41 39.6 [28.8-53.6] 0.89 1.52 0.59

Phe 65.4 [39.3-83.7] 25.4 [21.2-40.6] -61 38.8 [25.7-55.3] 0.59 1.53 0.39

Tyr 156 [111-229] 80.9 [63.5-124.5] -48 114 [81.5-153] 0.73 1.41 0.52

Lys 117 [80.5-203] 51.3 [41.3-87.5] -56 83.8 [55.1-131] 0.72 1.63 0.44

OH-Lys (D) 5.46 [3.10-7.29] 2.55 [1.63-3.76] -53 1.97 [1.95-4.04] 0.54 1.16 0.4

OH-Lys (L) 23.2 [15.0-32.1] 13.5 [9.88-18.2] -42 16.5 [12.4-21.98] 0.71 1.22 0.58

MML 6.72 [3.81-17.9] 3.06 [1.25-7.48] -54 4.47 [1.71-10.5] 0.67 1.46 0.46

CML 10.6 [6.13-18.1] 7.8 [4.89-11.9] -26 9.22 [6.05-12.3] 0.87 1.18 0.74

CEL 8.06 [5.33-12.4] 6.15 [4.4-7.78] -24 6.86 [4.56-9.21] 0.85 1.11 0.76

Furosine 1.63 [1.21-2.44] 0.89 [0.47-1.32] -45 0.905 [0.62-1.28] 0.56 1.02 0.55

Arg 36.3 [20.9-54.1] 16.5 [12.3-24.7] -53 28.9 [18.8-38.2] 0.82 1.75 0.47

GAA 281 [167-428] 166 [95.9-228.5] -41 125 [66.6-213] 0.44 0.75 0.5

hArg 0.97 [0.565-1.15] 0.64 [0.39-1.08] -34 0.67 [0.38-1.31] 0.69 1.05 0.66

ADMA 42.4 [26.7-56.4] 16.3 [12.5-23.1] -62 18.9 [12.6-28.1] 0.45 1.16 0.38

CMA 2.66 [1.77-3.45] 1.9 [1.39-2.47] -29 2.13 [1.53-2.95] 0.80 1.12 0.7

CEA 12.6 [7.65-26.9] 14 [8.05-26.5] +11 15.6 [10.7-22.9] 1.23 1.11 1.11

Trp 41.6 [31.7-77.2] 23.4 [16.8-37] -44 28 [16.2-42.7] 0.67 1.20 0.56

CEC 12 [5.15-17.8] 8.28 [4.26-15.7] -31 14.9 [8.69-24.4] 1.24 1.79 0.69

2SC 18.2 [12.2-28.2] 11.5 [8.05-14.8] -37 18.7 [13.6-23.2] 1.03 1.63 0.63

____________________________________________________________________________________________________________________________________________

**Table S2A.** KTR characteristics at baseline in tertiles of 24-h urinary lysine excretion

|  |  | **Tertile 1 (*n*=210)** | **Tertile 2 (*n*=210)** | **Tertile 3 (*n*=210)** | ***P* value** |
| --- | --- | --- | --- | --- | --- |
|  | Lysine excretion (µmol/24 h) | 47 [33 - 55] | 84 [74 - 96] | 170 [131 - 263] | <0.001 |
| **Clinical characteristics** | |  |  |  |  |
|  | Female sex, *n* (%) | 117 (55.7) | 81 (38.6) | 65 (31.0) | <0.001 |
|  | Age, y | 54.4 (12.0) | 53.9 (12.9) | 50.8 (13.1) | 0.007 |
|  | Primary renal disease, *n* (%) |  |  |  | 0.216 |
|  | Unknown | 28 (13.3) | 36 (17.1) | 29 (13.8) |  |
|  | Glomerulonephritis | 53 (25.2) | 57 (27.1) | 52 (24.8) |  |
|  | Interstitial nephritis | 25 (11.9) | 27 (12.9) | 28 (13.3) |  |
|  | Cystic kidney disease | 51 (24.3) | 41 (19.5) | 39 (18.6) |  |
|  | Other congenital/hereditary disease | 9 (4.3) | 16 (7.6) | 9 (4.3) |  |
|  | Renal vascular disease | 8 (3.8) | 8 (3.8) | 20 (9.5) |  |
|  | Diabetic nephropathy | 15 (7.1) | 6 (2.9) | 12 (5.7) |  |
|  | Other multisystem diseases | 16 (7.6) | 15 (7.1) | 13 (6.2) |  |
|  | Other | 5 (2.4) | 4 (1.9) | 8 (3.8) |  |
|  | Height, cm | 172 (9) | 174 (9) | 175 (10) | <0.001 |
|  | Weight, kg | 76 (17) | 82 (16) | 85 (16) | <0.001 |
|  | Body surface area, m^2^ | 1.88 (0.22) | 1.96 (0.20) | 2.00 (0.22) | <0.001 |
|  | Body mass index, kg/m^2^ | 25.7 (4.9) | 26.9 (4.8) | 27.6 (4.7) | 0.001 |
|  | Systolic blood pressure, mmHg | 135 (18) | 135 (17) | 139 (17) | 0.025 |
|  | Diabetes, *n* (%) | 47 (22.4) | 46 (21.9) | 59 (28.1) | 0.256 |
|  | History of cardiovascular disease, *n* (%) | 53 (25.2) | 57 (27.1) | 50 (23.8) | 0.733 |
|  | Smoking status, *n* (%) |  |  |  | 0.005 |
|  | Never | 70 (34.8) | 82 (41.6) | 85 (44.3) |  |
|  | History of smoking | 99 (49.3) | 103 (52.3) | 78 (40.6) |  |
|  | Current smoking | 32 (15.9) | 12 (6.1) | 29 (15.1) |  |
|  | Pre-emptive transplantation, *n* (%) | 34 (16.2) | 35 (16.7) | 35 (16.7) | 0.989 |
|  | Duration of dialysis, months | 25 [4 to 51] | 25 [6 to 45] | 22 [0 to 46] | 0.461 |
|  | Time after transplantation, y | 6.1 [1.9 to 12.8] | 5.2 [1.4 to 11.0] | 4.3 [1.4 to 9.0] | 0.013 |
|  | History of rejection, *n* (%) | 57 (27.1) | 53 (25.2) | 51 (24.3) | 0.792 |
|  | History of delayed graft function, *n* (%) | 17 (8.1) | 13 (6.2) | 18 (8.6) | 0.623 |
|  | Anti-HLA Class II antibodies, *n* (%) | 46 (21.9) | 28 (13.3) | 32 (15.2) | 0.048 |
|  | Donor age, y | 43 (15) | 44 (16) | 44 (15) | 0.891 |
|  | Living donor, *n* (%) | 70 (33.3) | 67 (31.9) | 84 (40.0) | 0.179 |
|  | Cold ischemia time, h | 17 [3 to 23] | 16 [3 to 21] | 12 [3 to 18] | 0.001 |
| **Laboratory measurements** | |  |  |  |  |
|  | Hemoglobin, g/dL | 7.9 (1.1) | 8.3 (1.0) | 8.5 (1.1) | <0.001 |
|  | Sodium mmol/L | 140.4 (3.1) | 141.0 (2.5) | 141.4 (2.6) | 0.001 |
|  | Potassium, mmol/L | 4.0 (0.5) | 4.0 (0.5) | 3.9 (0.5) | 0.276 |
|  | Creatinine, µmol/L | 135 [105 to 170] | 123 [101 to 161] | 116 [95 to 152] | 0.006 |
|  | Cystatin C, mg/L | 42 (18) | 45 (20) | 49 (18) | <0.001 |
|  | eGFR, mL/min/1.73 m^2^ | 39 (17) | 45 (17) | 51 (20) | <0.001 |
|  | Urea, mmol/L | 10.7 [8.0 to 15.7] | 9.5 [7.0 to 13.3] | 8.5 [6.8 to 11.4] | <0.001 |
|  | Uric Acid, mmol/L | 0.46 (0.12) | 0.43 (0.12) | 0.40 (0.10) | <0.001 |
|  | Triglycerides, mmol/L | 1.7 [1.3 to 2.3] | 1.7 [1.2 to 2.4] | 1.7 [1.2 to 2.2] | 0.995 |
|  | HDL cholesterol, mmol/L | 1.3 [1.1 to 1.8] | 1.3 [1.1 to 1.6] | 1.3 [1.0 to 1.6] | 0.050 |
|  | LDL cholesterol, mmol/L | 2.9 (0.9) | 3.0 (1.0) | 2.9 (0.9) | 0.554 |
|  | HbA1c, % | 5.8 [5.5 to 6.1] | 5.8 [5.5 to 6.3] | 5.8 [5.5 to 6.3] | 0.420 |
|  | Leukocyte count, 10^9^/L | 8.1 (3.1) | 8.1 (2.4) | 8.2 (2.4) | 0.884 |
|  | hs-CRP, mg/L | 1.5 [0.8 to 4.1] | 1.5 [0.6 to 5.2] | 1.7 [0.7 to 4.8] | 0.924 |
|  | Albumin, g/L | 42.6 (2.8) | 42.9 (3.3) | 43.4 (2.8) | 0.026 |
|  | Urinary protein excretion, g/24 h | 0.4 (0.7) | 0.4 (0.9) | 0.5 (0.8) | 0.302 |
|  | Urinary sodium excretion, mmol/24 h | 133 (542) | 157 (59) | 184 (64) | <0.001 |
|  | Urinary urea excretion, mmol/24 h | 341 (101) | 398 (97) | 436 (122) | <0.001 |
|  | Urinary creatinine excretion, mmol/24 h | 10.1 (3.1) | 12.0 (3.2) | 13.2 (3.5) | <0.001 |
| **Medication** | |  |  |  |  |
|  | Antihypertensive drugs, *n* (%) | 188 (89.5) | 180 (85.7) | 187 (89.0) | 0.422 |
|  | Prednisolone, *n* (%) | 208 (99.0) | 210 (100.0) | 209 (99.5) | 0.366 |
|  | Calcineurin inhibitor, *n* (%) | 127 (60.5) | 113 (53.8) | 119 (56.7) | 0.384 |
|  | Proliferation inhibitor, *n* (%) | 170 (81.0) | 188 (89.5) | 179 (85.2) | 0.047 |
|  | mTOR inhibitor, *n* (%) | 2 (1.0) | 6 (2.9) | 12 (5.7) | 0.020 |

Tertile 1: Lysine excretion <62.5 µmol/24 h, tertile 2: lysine excretion between 62.5 and 110.0 µmol/24 h, tertile 3: lysine excretion >110.0 µmol/24 h.

Normally distributed data are presented as mean ± standard deviation, skewed data as median [interquartile range], and categorical data as number (valid percentage).

Diabetes was defined according to the American Diabetes Association criteria. Data on smoking status was missing in 45 patients (7.1%), data on donor age was missing in 16 patients (2.5%), data on HbA1c was missing in 23 patients (3.7%), and data on hs-CRP was missing in 35 patients (5.6%).

All other variables had missing data for ≤10 patients.

Abbreviations: eGFR, estimated glomerular filtration rate as calculated using the creatinine and cystatin C-based CKD-EPI formula; hs-CRP, high-sensitivity C-reactive protein

**Table S2B** KTR characteristics at baseline in tertiles of 24-h urinary carboxymethyllysine (CML) excretion

|  |  | **Tertile 1 (*n*=210)** | **Tertile 2 (*n*=210)** | **Tertile 3 (*n*=210)** | ***P* value** |
| --- | --- | --- | --- | --- | --- |
|  | CML excretion (µmol/24 h) | 4.5 [2.6 - 6.1] | 9.2 [8.3 - 10.2] | 14.3 [12.2 - 17.3] | <0.001 |
| **Clinical characteristics** | |  |  |  |  |
|  | Female sex, *n* (%) | 118 (56.2) | 97 (46.2) | 48 (22.9) | <0.001 |
|  | Age, y | 53 (13) | 54 (13) | 52 (13) | 0.623 |
|  | Primary renal disease, *n* (%) |  |  |  | 0.998 |
|  | Unknown | 33 (15.7) | 31 (14.8) | 29 (13.8) |  |
|  | Glomerulonephritis | 53 (25.2) | 55 (26.2) | 54 (25.7) |  |
|  | Interstitial nephritis | 27 (12.9) | 26 (12.4) | 27 (12.9) |  |
|  | Cystic kidney disease | 46 (21.9) | 46 (21.9) | 39 (18.6) |  |
|  | Other congenital/hereditary disease | 11 (5.2) | 13 (6.2) | 10 (4.8) |  |
|  | Renal vascular disease | 9 (4.3) | 12 (5.7) | 15 (7.1) |  |
|  | Diabetic nephropathy | 11 (5.2) | 10 (4.8) | 12 (5.7) |  |
|  | Other multisystem diseases | 15 (7.1) | 12 (5.7) | 17 (8.1) |  |
|  | Other | 5 (2.4) | 5 (2.4) | 7 (3.3) |  |
|  | Height, cm | 171 (10) | 174 (9) | 177 (9) | <0.001 |
|  | Weight, kg | 77 (17) | 80 (16) | 86 (16) | <0.001 |
|  | Body surface area, m^2^ | 1.88 (0.22) | 1.94 (0.20) | 2.02 (0.21) | <0.001 |
|  | Body mass index, kg/m^2^ | 26.1 (5.0) | 26.5 (4.9) | 27.5 (4.7) | 0.010 |
|  | Systolic blood pressure, mmHg | 134 (18) | 135 (17) | 138 (17) | 0.040 |
|  | Diabetes, *n* (%) | 45 (21.4) | 46 (21.9) | 61 (29.0) | 0.124 |
|  | History of cardiovascular disease, *n* (%) | 43 (20.5) | 62 (29.5) | 55 (26.2) | 0.098 |
|  | Smoking status, *n* (%) |  |  |  | 0.385 |
|  | Never | 76 (37.4) | 80 (41.5) | 81 (41.8) |  |
|  | History of smoking | 101 (49.8) | 95 (49.2) | 84 (43.3) |  |
|  | Current smoking | 26 (12.8) | 18 (9.3) | 29 (14.9) |  |
|  | Pre-emptive transplantation, *n* (%) | 37 (17.6) | 39 (18.6) | 28 (13.3) | 0.305 |
|  | Duration of dialysis, months | 25 [4 to 50] | 24 [1 to 47] | 24 [8 to 46] | 0.791 |
|  | Time after transplantation, y | 6.0 [2.2 to 12.5] | 5.1 [1.7 to 10.2] | 4.5 [1.3 to 10.0] | 0.069 |
|  | History of rejection, *n* (%) | 57 (27.1) | 57 (27.1) | 47 (22.4) | 0.434 |
|  | History of delayed graft function, *n* (%) | 16 (7.6) | 11 (5.2) | 21 (10.0) | 0.184 |
|  | Anti-HLA Class II antibodies, *n* (%) | 49 (23.3) | 31 (14.8) | 26 (12.4) | 0.007 |
|  | Donor age, y | 43 (15) | 44 (15) | 44 (15) | 0.600 |
|  | Living donor, *n* (%) | 73 (34.8) | 74 (35.2) | 74 (35.2) | 0.993 |
|  | Cold ischemia time, h | 16 [3 to 23] | 15 [3 to 21] | 14 [3 to 19] | 0.250 |
| **Laboratory measurements** | |  |  |  |  |
|  | Hemoglobin, g/dL | 7.9 (1.1) | 8.2 (1.1) | 8.5 (1.0) | <0.001 |
|  | Sodium mmol/L | 140.2 (3.0) | 141.0 (2.7) | 141.7 (2.4) | <0.001 |
|  | Potassium, mmol/L | 4.0 (0.5) | 4.0 (0.5) | 4.0 (0.4) | 0.928 |
|  | Creatinine, µmol/L | 127 [103 to 159] | 126 [101 to 168] | 120 [98 to 153] | 0.224 |
|  | Cystatin C, mg/L | 2.0 (0.8) | 1.9 (0.9) | 1.7 (0.7) | 0.003 |
|  | eGFR, mL/min/1.73 m^2^ | 42 (18) | 45 (20) | 49 (18) | 0.001 |
|  | Urea, mmol/L | 9.5 [7.1 to 13.6] | 10.1 [7.3 to 14.1] | 9.1 [7.2 to 11.6] | 0.085 |
|  | Uric acid, mmol/L | 0.44 (0.12) | 0.43 (0.12) | 0.42 (0.11) | 0.435 |
|  | Triglycerides, mmol/L | 1.6 [1.3 to 2.3] | 1.7 [1.2 to 2.2] | 1.7 [1.3 to 2.4] | 0.648 |
|  | HDL cholesterol, mmol/L | 1.3 [1.1 to 1.7] | 1.3 [1.1 to 1.7] | 1.2 [1.1 to 1.5] | 0.080 |
|  | LDL cholesterol, mmol/L | 3.0 (0.9) | 2.9 (1.0) | 3.0 (0.9) | 0.767 |
|  | HbA1c, % | 5.8 [5.5 to 6.0] | 5.7 [5.5 to 6.1] | 6.0 [5.6 to 6.4] | 0.001 |
|  | Leukocyte count, 10^9^/L | 8.0 (2.8) | 8.0 (2.8) | 8.4 (2.3) | 0.192 |
|  | hs-CRP, mg/L | 1.6 [0.7 to 4.2] | 1.8 [0.8 to 4.9] | 1.4 [0.6 to 4.3] | 0.262 |
|  | Albumin, g/L | 42.8 (3.3) | 42.8 (2.9) | 43.3 (2.6) | 0.100 |
|  | Urinary protein excretion, g/24 h | 0.48 (1.06) | 0.34 (0.59) | 0.39 (0.69) | 0.210 |
|  | Urinary sodium excretion, mmol/24 h | 131 (56) | 153 (55) | 190 (63) | <0.001 |
|  | Urinary urea excretion, mmol/24 h | 343 (108) | 389 (98) | 444 (113) | <0.001 |
|  | Urinary creatinine excretion, mmol/24 h | 10.3 (3.3) | 11.6 (2.9) | 13.5 (3.5) | <0.001 |
| **Medication** | |  |  |  |  |
|  | Antihypertensive drugs, *n* (%) | 189 (90.0) | 181 (86.2) | 185 (88.1) | 0.484 |
|  | Prednisolone, *n* (%) | 207 (98.6) | 210 (100.0) | 210 (100.0) | 0.049 |
|  | Calcineurin inhibitor, *n* (%) | 115 (54.8) | 117 (55.7) | 127 (60.5) | 0.448 |
|  | Proliferation inhibitor, *n* (%) | 185 (88.1) | 178 (84.8) | 174 (82.9) | 0.309 |
|  | mTOR inhibitor, *n* (%) | 5 (2.4) | 7 (3.3) | 8 (3.8) | 0.697 |

Tertile 1: CML excretion <7.45 µmol/24 h, tertile 2: CML excretion between 7.45 and 11.10 µmol/24 h, tertile 3: CML excretion >11.10 µmol/24 h.

Normally distributed data are presented as mean ± standard deviation, skewed data as median [interquartile range], and categorical data as number (valid percentage).

Diabetes was defined according to the American Diabetes Association criteria.

Data on smoking status was missing in 45 patients (7.1%), data on donor age was missing in 16 patients (2.5%), data on HbA1c was missing in 23 patients (3.7%), and data on hs-CRP was missing in 35 patients (5.6%). All other variables had missing data for ≤10 patients.

Abbreviations: eGFR, estimated glomerular filtration rate as calculated using the creatinine and cystatin C -based CKD-EPI formula; hs-CRP, high-sensitivity C-reactive protein

**Table S2C** KTR characteristics at baseline in tertiles of 24-h urinary furosine excretion

|  |  | **Tertile 1**  **(*n* = 210)** | **Tertile 2**  **(*n* = 210)** | **Tertile 3**  **(*n* = 210)** | ***P*-value** |
| --- | --- | --- | --- | --- | --- |
|  | Furosine excretion (µmol/24 h) | 0.5 [0.3 - 0.6] | 0.9 [0.8 - 1.0] | 1.6 [1.3 - 1.9] | <0.001 |
| **Clinical characteristics** | |  |  |  |  |
|  | Female sex, *n* (%) | 106 (50.5) | 97 (46.2) | 60 (28.6) | <0.001 |
|  | Age, y | 55 (12) | 53 (13) | 51 (13) | 0.013 |
|  | Primary renal disease, *n* (%) |  |  |  | 0.116 |
|  | Unknown | 32 (15.2) | 29 (13.8) | 32 (15.2) |  |
|  | Glomerulonephritis | 45 (21.4) | 62 (29.5) | 55 (26.2) |  |
|  | Interstitial nephritis | 29 (13.8) | 24 (11.4) | 27 (12.9) |  |
|  | Cystic kidney disease | 48 (22.9) | 47 (22.4) | 36 (17.1) |  |
|  | Other congenital/hereditary disease | 10 (4.8) | 12 (5.7) | 12 (5.7) |  |
|  | Renal vascular disease | 10 (4.8) | 14 (6.7) | 12 (5.7) |  |
|  | Diabetic nephropathy | 16 (7.6) | 11 (5.2) | 6 (2.9) |  |
|  | Other multisystem diseases | 16 (7.6) | 5 (2.4) | 23 (11.0) |  |
|  | Other | 4 (1.9) | 6 (2.9) | 7 (3.3) |  |
|  | Height, cm | 173 (9) | 173 (9) | 176 (10) | 0.001 |
|  | Weight, kg | 79 (17) | 80 (17) | 84(16) | 0.010 |
|  | Body surface area, m^2^ | 1.92 (0.22) | 1.93 (0.22) | 1.99 (0.21) | 0.001 |
|  | Body mass index, kg/m^2^ | 26.5 (5.0) | 26.7 (4.9) | 27.0 (4.7) | 0.490 |
|  | Systolic blood pressure, mmHg | 136 (19) | 136 (17) | 136 (16) | 0.970 |
|  | Diabetes, *n* (%) | 48 (22.9) | 52 (24.8) | 52 (24.8) | 0.870 |
|  | History of cardiovascular disease, *n* (%) | 59 (28.1) | 60 (28.6) | 41 (19.5) | 0.056 |
|  | Smoking status, *n* (%) |  |  |  | 0.118 |
|  | Never | 82 (40.8) | 72 (36.9) | 83 (42.8) |  |
|  | History of smoking | 92 (45.8) | 106 (54.4) | 82 (42.3) |  |
|  | Current smoking | 27 (13.4) | 17 (8.7) | 29 (14.9) |  |
|  | Pre-emptive transplantation, *n* (%) | 32 (15.2) | 37 (17.6) | 35 (16.7) | 0.803 |
|  | Duration of dialysis, months | 26 [5 to 50] | 23 [0 to 46] | 24 [7 to 47] | 0.374 |
|  | Time after transplantation, y | 5.9 [1.7 to 12.5] | 5.1 [1.7 to 11.2] | 4.7 [1.5 to 9.4] | 0.122 |
|  | History of rejection, *n* (%) | 57 (27.1) | 57 (27.1) | 47 (22.4) | 0.434 |
|  | History of delayed graft function, *n* (%) | 14 (6.7) | 18 (8.6) | 16 (7.6) | 0.763 |
|  | Anti-HLA Class II antibodies, *n* (%) | 39 (18.6) | 39 (18.6) | 28 (13.3) | 0.253 |
|  | Donor age, y | 44 (15) | 44 (15) | 42 (15) | 0.457 |
|  | Living donor, *n* (%) | 65 (31.0) | 72 (34.3) | 84 (40.0) | 0.145 |
|  | Cold ischemia time, h | 16 [3 to 22] | 16 [3 to 21] | 13 [3 to 19] | 0.009 |
| **Laboratory measurements** | |  |  |  |  |
|  | Hemoglobin, g/dL | 7.8 (1.1) | 8.2 (1.1) | 8.6 (0.9) | <0.001 |
|  | Sodium, mmol/L | 140.3 (3.1) | 141.2 (2.6) | 141.3 (2.4) | <0.001 |
|  | Potassium, mmol/L | 4.0 (0.5) | 3.9 (0.5) | 4.0 (0.4) | 0.359 |
|  | Creatinine, µmol/L | 141 [108 to 186] | 126 [102 to 163] | 113 [94 to 139] | <0.001 |
|  | Cystatin C, mg/L | 2.2 (1.0) | 1.8 (0.7) | 1.5 (0.5) | <0.001 |
|  | eGFR, mL/min/1.73 m^2^ | 38 (17) | 44 (17) | 55 (18) | <0.001 |
|  | Urea, mmol/L | 11.2 [8.3 to 16.1] | 9.5 [7.0 to 13.4] | 8.2 [6.8 to 10.5] | <0.001 |
|  | Uric acid, mmol/L | 0.46 (0.13) | 0.43 (0.11) | 0.41 (0.10) | <0.001 |
|  | Triglycerides, mmol/L | 1.7 [1.3 to 2.3] | 1.7 [1.2 to 2.5] | 1.7 [1.2 to 2.2] | 0.717 |
|  | HDL cholesterol, mmol/L | 1.3 [1.0 to 1.7] | 1.3 [1.0 to 1.6] | 1.3 [1.1 to 1.6] | 0.888 |
|  | LDL cholesterol, mmol/L | 2.9 (1.0) | 3.0 (0.9) | 3.0 (0.9) | 0.205 |
|  | HbA1c, % | 5.8 [5.5 to 6.1] | 5.8 [5.5 to 6.3] | 5.8 [5.6 to 6.3] | 0.166 |
|  | Leukocyte count, 10^9^/L | 8.0 (2.6) | 8.4 (2.9) | 8.1 (2.3) | 0.280 |
|  | hs-CRP, mg/L | 1.6 [0.7 to 5.4] | 1.9 [0.9 to 5.3] | 1.3 [0.6 to 3.2] | 0.004 |
|  | Albumin, g/L | 42.4 (3.2) | 42.9 (2.8) | 43.6 (2.8) | <0.001 |
|  | Urinary protein excretion, g/24 h | 0.5 (1.0) | 0.4 (0.7) | 0.3 (0.6) | 0.107 |
|  | Urinary sodium excretion, mmol/24 h | 135.07 (56.61) | 153.43 (55.68) | 185.73 (65.45) | <0.001 |
|  | Urinary urea excretion, mmol/24 h | 351.18 (104.45) | 379.27 (100.87) | 444.51 (116.58) | <0.001 |
|  | Urinary creatinine excretion, mmol/24 h | 10.47 (3.24) | 11.34 (3.07) | 13.49 (3.42) | <0.001 |
| **Medication** | |  |  |  |  |
|  | Antihypertensive drugs, *n* (%) | 191 (91.0) | 188 (89.5) | 176 (83.8) | 0.057 |
|  | Prednisolone, *n* (%) | 208 (99.0) | 209 (99.5) | 210 (100.0) | 0.366 |
|  | Calcineurin inhibitor, *n* (%) | 123 (58.6) | 117 (55.7) | 119 (56.7) | 0.834 |
|  | Proliferation inhibitor, *n* (%) | 176 (83.8) | 180 (85.7) | 181 (86.2) | 0.767 |
|  | mTOR inhibitor, *n* (%) | 4 (1.9) | 8 (3.8) | 8 (3.8) | 0.438 |

Tertile 1: furosine excretion <0.70 µmol/24 h, tertile 2: furosine excretion between 0.70 and 1.12 µmol/24h, tertile 3: furosine excretion >1.12 µmol/24 h.

Normally distributed data are presented as mean ± standard deviation, skewed data as median [interquartile range], and categorical data as number (valid percentage).

Diabetes was defined according to the American Diabetes Association criteria.

Data on smoking status was missing in 45 patients (7.1%), data on donor age was missing in 16 patients (2.5%), data on HbA1c was missing in 23 patients (3.7%), and data on hs-CRP was missing in 35 patients (5.6%). All other variables had missing data for ≤10 patients.

Abbreviations: eGFR, estimated glomerular filtration rate as calculated using the creatinine and cystatin C -based CKD-EPI formula; hs-CRP, high-sensitivity C-reactive protein

| **Table S3.** Causal pathway analyses on the association of urinary excretion rates of CML and furosine with all-cause mortality, cardiovascular mortality, and non-cardiovascular mortality | | | | | | | | | | |
| --- | --- | --- | --- | --- | --- | --- | --- | --- | --- | --- |
|  | | **All-cause mortality** | | |  | **Cardiovascular mortality** | |  | **Non-cardiovascular mortality** | |
| ***CML*** | | **HR per SD increase [95% CI]** | | ***P*** |  | **HR per SD increase  [95% CI]** | ***P*** |  | **HR per SD increase [95% CI]** | ***P*** |
|  | **Model 1** | 0.78 [0.64 – 0.96] | 0.02 | |  | 0.76 [0.56 – 1.04] | 0.09 |  | 0.80 [0.61 – 1.06] | 0.11 |
|  | **Model 2** | 0.81 [0.65 – 0.99] | 0.05 | |  | 0.77 [0.56 – 1.05] | 0.10 |  | 0.85 [0.64 – 1.13] | 0.27 |
|  | **Model 3** | 0.83 [0.67 – 1.03] | 0.10 | |  | 0.79 [0.57 – 1.10] | 0.16 |  | 0.87 [0.64 – 1.16] | 0.34 |
|  | **Model 4** | 0.87 [0.70 – 1.09] | 0.23 | |  | 0.84 [0.60 – 1.18] | 0.31 |  | 0.90 [0.67 – 1.21] | 0.48 |
| ***Furosine*** | | | | | | | | | | |
|  | **Model 1** | 0.75 [0.59 – 0.96] | 0.03 | |  | 0.59 [0.39 – 0.90] | 0.01 |  | 0.89 [0.65 – 1.21] | 0.44 |
|  | **Model 2** | 0.79 [0.61 – 1.02] | 0.07 | |  | 0.59 [0.39 – 0.90] | 0.01 |  | 0.97 [0.70 – 1.34] | 0.86 |
|  | **Model 3** | 0.84 [0.65 – 1.10] | 0.21 | |  | 0.63 [0.41 – 0.97] | 0.04 |  | 1.03 [0.74 – 1.44] | 0.84 |
|  | **Model 4** | 0.90 [0.69 – 1.17] | 0.42 | |  | 0.68 [0.44 – 1.06] | 0.09 |  | 1.07 [0.77 – 1.47] | 0.69 |
| Model 1 | | Adjusted for age, sex, BMI, eGFR and proteinuria. | | | | | | | | |
| Model 2 | | Model 1, with additional adjustment for urinary sodium excretion, reflecting salt intake | | | | | | | | |
| Model 3 | | Model 1, with additional adjustment for urinary urea excretion, reflecting protein intake | | | | | | | | |
| Model 4 | | Model 1, with additional adjustment for urinary creatinine excretion, reflecting muscle mass | | | | | | | | |
| eGFR was calculated according to the Chronic Kidney Disease Epidemiology formula with plasma creatinine and plasma cystatin C. Proportional hazards assumption was not violated in any of the models. | | | | | | | | | | |
